# FireProt^DB^: database of manually curated protein stability data

**DOI:** 10.1093/nar/gkaa981

**Published:** 2020-11-09

**Authors:** Jan Stourac, Juraj Dubrava, Milos Musil, Jana Horackova, Jiri Damborsky, Stanislav Mazurenko, David Bednar

**Affiliations:** Loschmidt Laboratories, Department of Experimental Biology and RECETOX, Masaryk University, Brno, Czech Republic; International Clinical Research Center, St. Anne's University Hospital Brno, Brno, Czech Republic; Loschmidt Laboratories, Department of Experimental Biology and RECETOX, Masaryk University, Brno, Czech Republic; Department of Information Systems, Faculty of Information Technology, Brno University of Technology, Brno, Czech Republic; Loschmidt Laboratories, Department of Experimental Biology and RECETOX, Masaryk University, Brno, Czech Republic; International Clinical Research Center, St. Anne's University Hospital Brno, Brno, Czech Republic; Department of Information Systems, Faculty of Information Technology, Brno University of Technology, Brno, Czech Republic; Loschmidt Laboratories, Department of Experimental Biology and RECETOX, Masaryk University, Brno, Czech Republic; Loschmidt Laboratories, Department of Experimental Biology and RECETOX, Masaryk University, Brno, Czech Republic; International Clinical Research Center, St. Anne's University Hospital Brno, Brno, Czech Republic; Loschmidt Laboratories, Department of Experimental Biology and RECETOX, Masaryk University, Brno, Czech Republic; Loschmidt Laboratories, Department of Experimental Biology and RECETOX, Masaryk University, Brno, Czech Republic; International Clinical Research Center, St. Anne's University Hospital Brno, Brno, Czech Republic

## Abstract

The majority of naturally occurring proteins have evolved to function under mild conditions inside the living organisms. One of the critical obstacles for the use of proteins in biotechnological applications is their insufficient stability at elevated temperatures or in the presence of salts. Since experimental screening for stabilizing mutations is typically laborious and expensive, *in silico* predictors are often used for narrowing down the mutational landscape. The recent advances in machine learning and artificial intelligence further facilitate the development of such computational tools. However, the accuracy of these predictors strongly depends on the quality and amount of data used for training and testing, which have often been reported as the current bottleneck of the approach. To address this problem, we present a novel database of experimental thermostability data for single-point mutants FireProt^DB^. The database combines the published datasets, data extracted manually from the recent literature, and the data collected in our laboratory. Its user interface is designed to facilitate both types of the expected use: (i) the interactive explorations of individual entries on the level of a protein or mutation and (ii) the construction of highly customized and machine learning-friendly datasets using advanced searching and filtering. The database is freely available at https://loschmidt.chemi.muni.cz/fireprotdb.

## INTRODUCTION

Proteins play essential roles in many biotechnological and biomedical applications, where they are often subjected to extreme environments, e.g. elevated temperatures or the presence of various salts. However, naturally occurring proteins have mostly evolved to function in the mild environmental conditions, and therefore their applicability is limited in the industrial applications. For this reason, protein engineers generally aim to improve protein stability, and thermostability is one of their primary targets (1) as it is correlated with serum survival time (2), half-life (3), expression yield (4) and activity in the presence of denaturants (5). A reliable assessment of the effect of a mutation on protein stability is often performed experimentally. Extensive experimental screening, however, is slow and costly, prompting the use of *in silico* approaches for the pre-selection of promising mutations. These methods are usually based on one of the three principles: (i) free energy calculations, (ii) phylogenetics or (iii) machine learning. With the recent advances in artificial intelligence, tool developers increasingly resort to the third group of methods. However, the accuracy of the machine learning-based predictors is still severely limited by the lack of high-quality data (6). Experimental characterizations are usually not capable of producing large amounts of data, and the majority of these measurements are scattered in the scientific literature. Thus, there is a strong demand for systematic collection, validation, and organization of such data in a database.

Two attempts have been made to establish a systematic and extensive collection of thermostability data so far. The first and largest database is the Thermodynamic Database for Proteins and Mutants–ProTherm (7). It was first released in 1999 with the aim to collect experimentally determined thermodynamic parameters for wild-type proteins and their mutants from the published literature. Its latest version contains >25 000 entries from 740 proteins, and it serves as the primary source of protein stability data for the development of new predictors. However, ProTherm was last updated in 2013 so the database is already out-of-date. Moreover, several critical issues have been reported, such as inaccurate annotations or wrong signs of values (6,8–10). This makes ProTherm even more difficult to use as time-demanding manual filtering and validation steps are required to confirm the values in the original articles. This manual filtering led to the construction of many different, often overlapping, subsets with corrected values and occasionally new data. Some of these derivative datasets were deposited to the VariBench database (11) without any attempts to reintegrate the changes into ProTherm or create an improved database. This changed in 2018 when ProtaBank (12) was released. This database aims to collect a wide range of protein engineering data such as thermostability, activity, expression, binding and several others. The developers imported all the data from ProTherm, yet they did not seem to perform any manual curation. Therefore, the critical issues listed above were not resolved. And while ProtaBank enriched the ProTherm data with recent experimental studies, the database does not offer any advanced searching and filtering capabilities, at least in its non-commercial version. This makes the data extraction and processing tedious by necessitating many manual steps and hindering the application of such data-driven methods as machine learning.

To overcome these limitations, we established the FireProt^DB^ database that holds manually curated thermostability data for single-point mutants. The database contains the data available in ProTherm, ProtaBank, and our extensive manual literature search. Its user-friendly interface allows easy and interactive browsing through the experimental data and provides links to the corresponding UniProt and PDB entries. Moreover, advanced searching and filtering capabilities, the ability to download the data in a simple table format, and meticulous labelling of data entries used for training and testing of published tools prompt the further application of machine learning.

## MATERIALS AND METHODS

### Database architecture and data model

The top-level entity of the FireProt^DB^ database is a unique protein sequence entry with the assigned UniProt ID (13). Protein sequences were preferred to structures due to the broader availability of the former. Each sequence is a string of amino acids in specified positions. Multiple mutations can be assigned to a single position, and each mutation can be evaluated by multiple measurements and derived values. The measurements represent the experimental values of the Gibbs free energy changes upon mutation (ΔΔ*G*) or changes in melting temperatures (Δ*T*_m_). The derived values stand for averages or medians of multiple measurements for a particular mutation. Each measurement is also accompanied by a curation flag that indicates whether the value was manually validated against the original publication to guarantee its correctness. Furthermore, each measurement and derived value can be assigned to multiple published datasets to promote accurate validation and benchmarking of computational tools.

From the structural point of view, each sequence can have one or more assigned biological units that denote biologically relevant quaternary structures of asymmetric units stored in the PDB database (14). For representative biological units, the HotSpot Wizard 3.0 (15) calculation was executed to compute additional sequential and structural annotations. These annotations can help with the analysis of selected mutations and serve as pre-calculated features applicable in machine learning models.

### Stability data acquisition and curation

**Figure 1.**
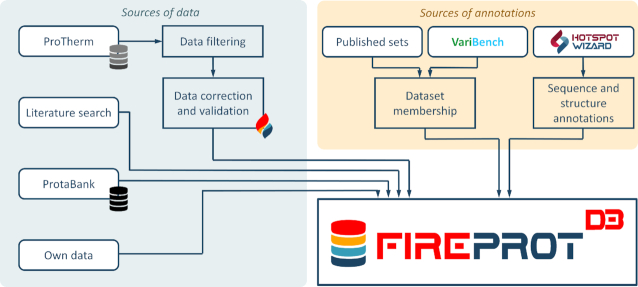
A schematic representation of the data comprising FireProt^DB^. The primary source of data is filtered ProTherm (7). The FireProt data subset (18) was manually curated, compared to the source publications, and marked with the ‘curated’ flag. The publications from ProtaBank (12) and manual literature search were also used to deposit the data. Each mutation in the deposited data was annotated according to its membership in the published datasets and those deposited on VariBench (11). The HotSpot Wizard 3.0 (15) annotation tool was applied to each protein entry with a known tertiary structure.

FireProt^DB^ is composed of the data from four sources: the ProTherm database, the ProtaBank database, manual mining of the scientific literature, and data collected in our laboratory (Figure 1). The primary data source was ProTherm. Due to the multiple problems mentioned in the introduction, we followed several filtering steps. In the first step, we retained only those entries that met the following four criteria: (i) they have a single-point mutation; (ii) the mutation is not an insertion or deletion; (iii) the protein has a SwissProt accession code and/or a PDB identifier; (iv) the entry includes a measured ΔΔ*G* and/or Δ*T*_m_. Secondly, we performed a validity check of SwissProt accession codes and updated obsolete entries. ProTherm references mutations by their structure index, i.e., the residue number in the structure, which in many cases does not match their sequence index, i.e. the position in the sequence. To overcome this issue, we used a similar approach as in PDBSWS (16): use the Needleman-Wunsch algorithm (17) to construct the global sequence alignment of sequences extracted from PDB and UniProt entries and map the mutations onto the UniProt sequences. In the next step, we confirmed that the reported wild-type amino acids are in the correct positions in the structures and unified the reported units. Finally, we matched the data with the manually curated entries in the FireProt dataset (18), updated the values, and marked them as ‘curated’.

In addition to ProTherm, we explored the studies reported in the ProtaBank database, extracted the thermostability data, and integrated them into our database. We also performed a manual literature search using stability-based keywords such as ‘protein stability’, ‘thermostability’, ‘free energy upon mutation’, ‘protein stabilization’. We mined the recent scientific articles reporting mutants with measured stability data and contacted the authors of the publications when the relevant data were not available in the article. All such entries were marked as ‘curated’ as we extracted them directly from the original publications. Finally, we reviewed the thermostability data collected in our lab throughout the last few years and added them to the database. We perform experimental protein characterization in our protein engineering projects on a regular basis, and measuring protein stability is an essential part of such characterization. In total, the three sources led to a significant enlargement of the data size by 62% in terms of all the entries. The number of curated entries more than doubled compared to the previously collected cleaned FireProt subset of ProTherm.

### Dataset assignment

In the second acquisition step, we collected 40 datasets from the VariBench database (11) and literature (18), which were used previously for training or testing of existing predictors. Since all these datasets are at least partially derived from ProTherm, we could label each measurement in FireProt^DB^ by its membership in the datasets. These labels are particularly useful for the comparison of new prediction models to the existing tools. This task is usually done by the performance evaluation of predictors on a dataset that is entirely independent of the training and test sets used for the development of the tools. Since the dataset construction is often laborious and consists of a manual data processing, the possibility to directly exclude the data present in given datasets significantly simplifies and speeds up the construction process.

### Calculation of additional annotations

To provide our users with a more advanced description of their proteins of interest, we enriched the database by several important sequence- and structure-related information. These calculations were performed by HotSpot Wizard 3.0 (15), which is currently the only tool capable of deriving all these features in a single calculation (19) and provides machine-readable results. HotSpot Wizard was executed on a representative biological unit of each protein and provided the annotations for a structure, such as the residues located in protein pockets and tunnels, and a sequence, such as catalytic residues, evolutionary conservation scores, back-to-consensus mutations, and correlated pairs. These annotations can be helpful for a better understanding of structure-function relationships as well as for generating features for machine learning.

## RESULTS

### Web interface

The web interface was designed for both types of expected users—protein chemists and software developers. Protein chemists are often looking for the thermostability evidence for their protein of interest, and they will benefit from its interactivity and details pages with additional information. Machine learning experts and bioinformaticians will be more interested in advanced filtering capabilities facilitating the process of construction of highly customized datasets for the training or assessment of various predictors. The entry point to the database is the search form, which allows browsing in two major ways: (i) a simple full-text search for querying the database using protein name, UniProt accession codes, PDB identifiers, protein names, publications, authors or organisms and (ii) an advanced search allowing the users to construct complex rules based on the relational algebra and all available database fields. The latter is one of the key features of FireProt^DB^ as it facilitates the construction of highly customized datasets needed for the development of new predictors.

Once the user clicks on the ‘Search’ button, they are redirected to the page with the result table. This table contains a list of available experiments, their basic annotations, and measured values. The table is paginated to eliminate possible performance issues and allows further interactive filtering of displayed values. The user can then easily export the search results in the CSV format using the ‘Export’ button at the top or the bottom of the page.

Clicking on a mutation name leads to a page with a more detailed view, showing all the data entries and datasets that include the selected mutation. Clicking on a protein name leads to a page providing the basic information such as UniProt accession code, organism and Enzyme Commission number, as well as detailed annotation of secondary structure, catalytic sites, natural variants and amino acid charges derived from UniProt database using interactive ProtVista tracks (20). This page also contains a list of all known biological units and a table with all experimental measurements.

### Search queries

**Figure 2.**
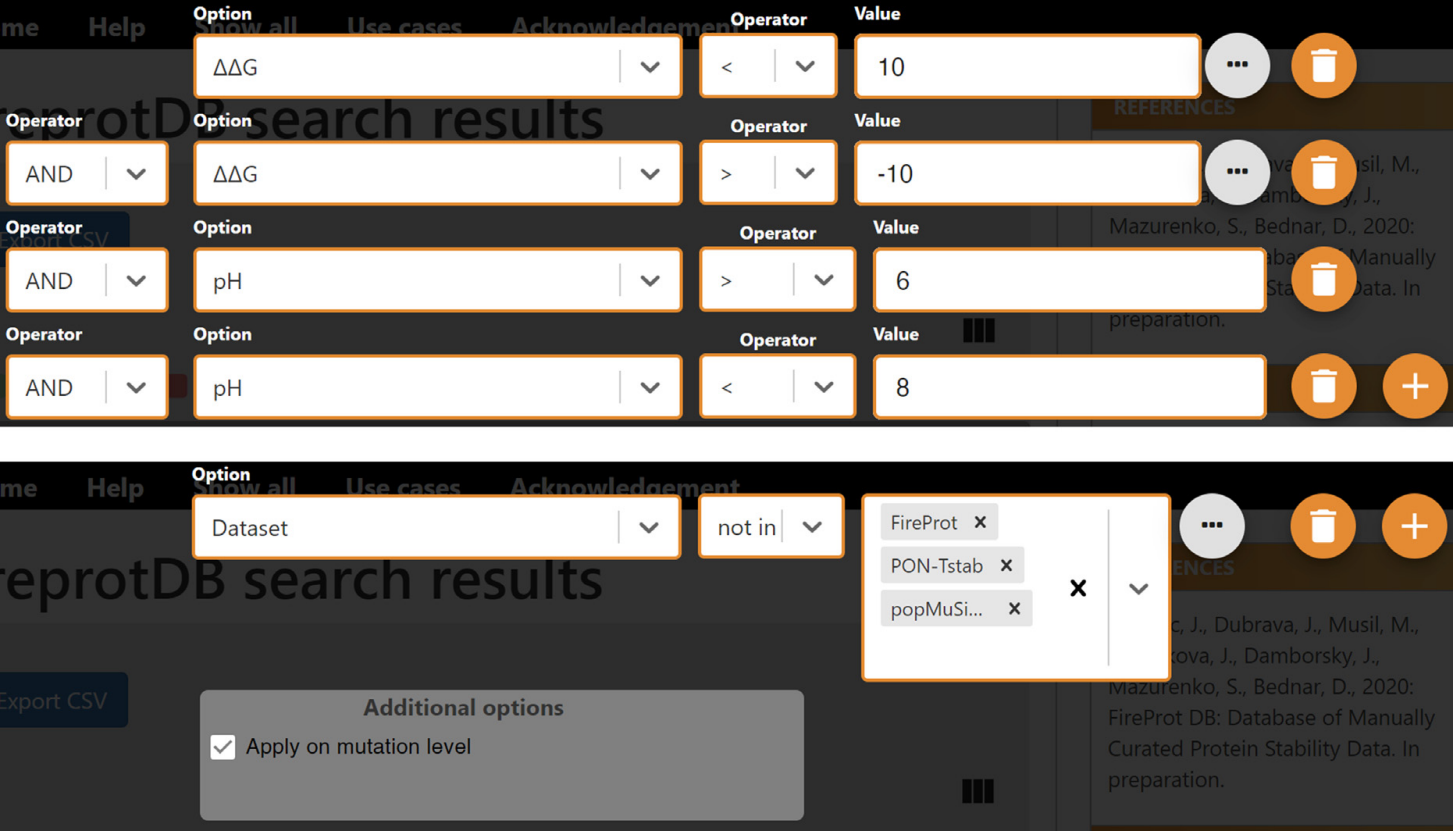
Examples of filtering protocols in FireProt^DB^. **Top**: The request filters out the data collected at extreme pH or with extreme ΔΔ*G* values, resulting in >3500 data points left. **Bottom**: An example of excluding all the mutations that appear in PopMuSiC, FireProt, or PON-Tstab datasets.

Several types of search queries may be of interest to the users. The first one relates to data filtering by values (10). Typically, software developers filter out the data collected at extreme pH (<6 or >8) due to changes in charged states for ionizable residues. The entries with large absolute ΔΔ*G* or Δ*T*_m_ are also sometimes excluded due to likely higher measurement errors, and also because dramatic changes to the stability may indicate significant structural alterations to the wild type, which may become a problem for structure-based features. The second type is relevant for benchmarking of a newly designed predictor against the existing tools or creating a meta predictor. In either case, one usually needs to derive a data subset that has not been used by the existing predictors for training. The main reason is the robust performance estimate, which is typically over-optimistic for these sets (6). Two corresponding examples of such filtering protocols are shown in Figure 2.

### Database dump

For the users requesting even higher control over the data and filtering capabilities, we offer the possibility to download the complete dump of the database in the SQL format. This data file can be easily imported to any modern MariaDB server, version 10.2, and higher. Since the database structure is complex and any custom query requires joining of multiple tables, the dump also contains a pre-defined view ‘mutation_experiments_summary’. The summary combines all the tables and provides the data in a similar structure as the CSV export from the user interface. This view or its definition can serve as a useful starting point for additional filtering or creating custom queries.

### Data statistics

**Figure 3.**
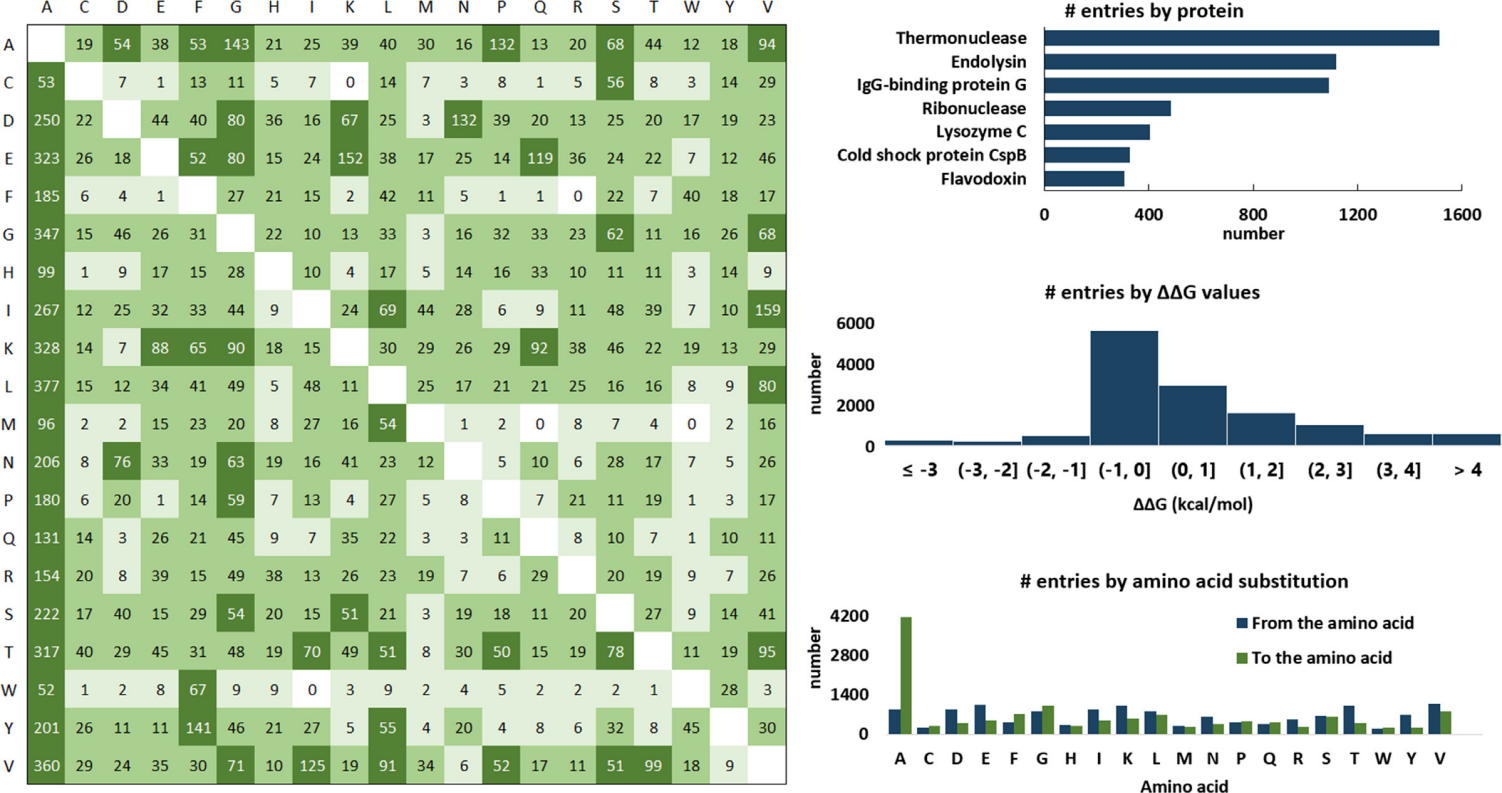
An overview of the data deposited to FireProt^DB^. **Left**: The table shows the total number of each substitution pair with the wild type amino acids in rows, mutant amino acids in columns, and the coloring according to the thresholds of 1 (light green), 10 (medium green) and 50 (dark green) entries for the corresponding substitution. **Right**: Histograms showing the top seven proteins by their UniProt IDs, the ΔΔ*G* values, and the cumulative number of amino acid substitutions.

Currently, FireProt^DB^ contains 13274 entries for 237 proteins (Figure 3), from which 8189 measurements originated from ProTherm. The remaining 5085 entries were added from our literature search (18%), publications from ProtaBank (28%), VariBench (53%), and our own records (1%). In total, 43% entries are destabilizing mutations (Δ*T*_m_←1 or ΔΔ*G* > 1 kcal/mol), 14% stabilizing (Δ*T*_m_ > 1 or ΔΔ*G*←1 kcal/mol), and 43% considered neutral (–1 ≤ Δ*T*_m_ ≤ 1 or – 1 ≤ ΔΔ*G* ≤ 1 kcal/mol). The database also includes annotations for 40 various published datasets derived from ProTherm, deposited to VariBench (11), or available in the corresponding articles and web servers. As far as enzymes are concerned, those collected in the database cover the first six EC classes, three of which by >40% on the second level.

## DISCUSSION

The availability of large high-quality datasets is one of the critical requirements for the advancement of machine learning-based *in silico* predictors. While some promising high-throughput experimental methods have been released recently (21,22), their validation is still ongoing, and protein stability experiments are still time-consuming and expensive. Building training and testing datasets is hindered by the data being hidden in the original articles, generating a strong demand for their systematic mining, collection, validation, and homogenization. The existing databases are not fulfilling all the requirements as ProTherm is outdated and contains incorrect data, and ProtaBank does not provide advanced search and export tools and is partly commercial.

FireProt^DB^ is a novel database for experimental thermostability data of protein single-point mutants. It consists of the data manually extracted from ProTherm, articles from ProtaBank, new data obtained by mining the recent literature, and the data collected in our laboratory. The database is accessible via a user-friendly graphical web interface allowing the users to search and browse the data interactively. Moreover, all the entries are annotated to indicate whether they belong to the already published datasets. These annotations, combined with the advanced searching and filtering capabilities, make FireProt^DB^ a valuable data resource for machine learning developers interested in constructing highly customized datasets.

In the future, we will improve our searching queries and employ automatic text-mining machine learning-based approaches (23–25) to accelerate literature mining and data collection, which will be followed by manual curation. We will also prepare an interactive form for data submissions by the users. Finally, we will extend the set of automatically generated features for mutations and add sequence similarity filtering to improve the data usability by the community of engineers applying machine learning to predict changes in protein stability.

## Notes

Website address: https://loschmidt.chemi.muni.cz/fireprotdb.
